# Cardiorespiratory fitness in individuals with type 2 diabetes mellitus: a systematic review and meta-analysis

**DOI:** 10.20945/2359-4292-2023-0040

**Published:** 2023-09-22

**Authors:** Aline Chagastelles Pinto de Macedo, Camila Wohlgemuth Schaan, Patricia Martins Bock, Mariana Brutto de Pinto, Cintia Ehlers Botton, Daniel Umpierre, Beatriz D. Schaan

**Affiliations:** 1 Universidade Federal do Rio Grande do Sul Porto Alegre RS Brasil Universidade Federal do Rio Grande do Sul, Programa de Pós-graduação em Ciências Médicas: Endocrinologia, Porto Alegre, RS, Brasil; 2 Laboratório de Atividade Física, Diabetes e Doença Cardiovascular Hospital de Clínicas de Porto Alegre Centro de Pesquisa Clínica Porto Alegre RS Brasil Laboratório de Atividade Física, Diabetes e Doença Cardiovascular (LADD), Centro de Pesquisa Clínica, Hospital de Clínicas de Porto Alegre, Porto Alegre, RS, Brasil; 3 Faculdades Integradas de Taquara Taquara RS Brasil Faculdades Integradas de Taquara, Taquara, RS, Brasil; 4 Universidade Federal do Rio Grande do Sul Porto Alegre RS Brasil Universidade Federal do Rio Grande do Sul, Porto Alegre, RS, Brasil; 5 Instituto de Avaliação de Tecnologia em Saúde Hospital de Clínicas de Porto Alegre Porto Alegre RS Brasil Instituto de Avaliação de Tecnologia em Saúde (IATS) – CNPq/Brasil, Hospital de Clínicas de Porto Alegre, Porto Alegre, RS, Brasil; 6 Universidade Federal do Ceará Instituto de Educação Física e Esportes Fortaleza CE Brasil Universidade Federal do Ceará, Instituto de Educação Física e Esportes, Fortaleza, CE, Brasil; 7 Universidade Federal do Ceará Fortaleza CE Brasil Programa de Mestrado em Fisioterapia e Funcionalidade, Universidade Federal do Ceará, Fortaleza, CE, Brasil

**Keywords:** Exercise tolerance, review, meta-analysis, diabetes mellitus

## Abstract

**Objective:**

To conduct a systematic review and meta-analysis assessing the cardiorespiratory fitness (CRF) among individuals with and without type 2 diabetes

**Materials and methods:**

The current review was registered in PROSPERO under the number CRD42018082718. MEDLINE, EMBASE, and Cochrane Library databases were searched from inception through February 2022. Eligibility criteria consisted of observational or interventional studies that evaluated CRF through cardiopulmonary exercise testing or six-minute walk test in individuals with type 2 diabetes compared with individuals without type 2 diabetes. For data extraction, we used baseline CRF assessments of randomized clinical trials or follow-up CRF assessments in observational studies. We performed a meta-analysis using maximal oxygen consumption (VO_2_max), and distance walked in the 6MWT as primary outcomes. They were extracted and expressed as mean differences (MDs) and 95% CIs between treatment and comparator groups. The meta-analysis was conducted using Review Manager (RevMan) software.

**Results:**

Out of 8,347 studies retrieved, 77 were included. Compared with individuals without type 2 diabetes, individuals with diabetes achieved a lower VO_2_max (−5.84 mL.kg^−1^.min^−1^, 95% CI −6.93, −4.76 mL.kg^−^^1^.min^−1^, p = <0.0001; I^2^ = 91%, p for heterogeneity < 0.0001), and a smaller distance walked in 6MWT (−93.30 meters, 95% CI −141.2, −45.4 meters, p > 0.0001; I^2^: 94%, p for heterogeneity < 0.0001).

**Conclusion:**

Type 2 diabetes was associated with lower cardiorespiratory fitness, as observed by lower VO_2_max on maximal tests, and smaller distance walked in 6MWT, however the quality of studies was low.

## INTRODUCTION

Cardiorespiratory fitness (CRF) appraises an individual's exercise capacity, it is directly linked to the integrated function of several body systems and may be a marker of total body health (1). Low CRF is associated with an increased risk of cardiovascular disease among patients with type 2 diabetes (2). Balducci and cols. (3) observed that increasing maximal oxygen consumption (VO_2_max) by approximately 2 mL.kg^−1^.min^−1^ can significantly reduce 10-year risk of coronary heart disease in these individuals. Moreover, a 9% lower relative risk of all-cause mortality was shown among adult men with VO_2_max of 1 mL.kg^−1^.min^−1^ higher (4). The annual cost savings per person were $5,193 in type 2 diabetes for each 1-metabolic equivalent (MET) higher fitness (5).

The cardiopulmonary exercise test – by gas analysis – is the gold standard assessment of CRF. It evaluates the VO_2_max or peak oxygen uptake (VO_2_ peak) during an incremental exercise test (6). Several protocols use a cycle ergometer or a treadmill (7), but these devices are expensive and require a trained team, being unfeasible in some situations such as population-based studies and in clinical practice. Therefore, other tests, such as six-minute walk test (6MWT), are also useful as they can estimate oxygen consumption (8).

Previous studies have shown controversial results when comparing CRF between individuals with and without diabetes: some showed comparable results (9–11), whereas others showed lower CRF in individuals with diabetes compared to those without diabetes (12–14). These differences could be methodological and derive from different protocols used for the evaluations. However, there are physiopathological mechanisms to justify the lower levels of exercise capacity observed among individuals with type 2 diabetes, which may occur from insulin action, mitochondrial dysfunction, skeletal muscle microvasculature, and cardiac dysfunction (15). Moreover, poor glycemic control can reduce CRF (15) because of diabetes itself or diabetes-associated sedentary behavior (16). Thus, it is essential to understand the magnitude of VO_2_max impairments observed in these individuals during planning of appropriate interventions to improve exercise performance and avoid increasing disability in this population. However, it is uncertain whether the magnitude of this difference and age, sex, body mass index (BMI), diabetes duration and control of the disease could negatively affect exercise capacity.

We aimed to conduct a systematic review with meta-analysis to summarize studies that assessed CRF measured by VO_2_ peak or VO_2_max in individuals with and without type 2 diabetes. We also evaluated the differences in distance walked in the 6MWT among them.

## MATERIALS AND METHODS

A systematic review and meta-analysis was conducted according to the Cochrane Handbook for Systematic Reviews of Interventions (17) and Preferred Reporting Items for Systematic Reviews and Meta-Analyses (PRISMA) guidelines (18). This review was registered in the international prospective register of systematic reviews (PROSPERO: CRD42018082718).

### Eligibility criteria

The eligibility criteria were as follows: 1) participants: adults with type 2 diabetes, > 18 years old; 2) outcomes: CRF measured by maximal exercise tests and expressed as VO_2_ (peak or maximal), or distance walked evaluated by the 6MWT; and 3) control group: individuals without type 2 diabetes; 4) study design: observational design (i.e., cohort or cross-sectional studies) and baseline data from quasi-experimental, randomized, or non-randomized clinical trials. Only studies in English, Portuguese, and Spanish were included. Studies were excluded if the participants had peripheral arterial disease, heart failure, chronic neurological diseases. Also, studies reporting that individuals without diabetes took any medication were excluded as well as studies when groups were matched by CRF.

### Outcomes definition

The primary outcome was VO_2_ (peak or maximal) measured by direct expired gas analysis. The secondary outcome was distance walked evaluated by the 6MWT.

### Databases and search strategy

Three electronic databases (i.e., PubMed/MEDLINE, EMBASE and Cochrane Library) were searched using a combination of MeSH headings, keywords and related entry terms, such as “type 2 diabetes” and “cardiorespiratory fitness”. The search strategies are presented in Supplementary File 1. Besides, the reference list of studies was manually searched. The search strategy was conducted from inception until December 2017, updated in March 2021 and February 2022.

### Selection process

Two pairs of authors (ACPM/MBP and PMB/CEB) independently evaluated the titles and abstracts of all studies based on eligibility criteria. All studies with abstracts lacking enough information regarding the eligibility criteria were included to full text evaluation. Finally, the full-text studies were evaluated by the same reviewers according to the inclusion and exclusion criteria and any disagreement between them was resolved by a third reviewer (DU).

### Data collection process

Data were extracted independently by two pairs of authors (ACPM/CWS and PMB/CEB) using a standardized and pre-tested data extraction form (Microsoft Excel). Missing data were requested to the authors by email (two out of seven requests were answered).

The information extracted from the included studies were sex, age, body mass index (BMI), medications, diabetes duration, hemoglobin A1c (HbA1c), physical activity level, exercise capacity test used and evaluated outcomes.

### Risk of bias and publication bias assessment

The risk of bias of the included studies was assessed by two pairs of authors (PMB/CEB and CWS/MBP), previously trained and qualified. The Newcastle-Ottawa Scale (NOS) version for cohort studies was adapted and used (19). The quality score was calculated by assessing three domains: selection of the study groups (0-3 points); comparability, which represent the quality of adjustment for confounding factors (0-2 points); evaluation of the outcomes of interest (0-3 points). The maximum score was eight and the classification of the studies were: (1) good quality: 2-3 points in the selection domain, 1-2 points in the comparability domain and 2-3 points in the outcome domain; (2) fair quality: 1 point in the selection domain, 1-2 points in the comparability domain and 1-2 points in the outcome domain; and (3) poor quality: 0 points in any domains. Disagreement between reviewers were resolved by consensus, and, in cases of persistent disagreement, the assessment was made by a third reviewer (ACPM).

Publication bias was assessed using a contour-enhanced funnel plot with each study effect size against the standard error of the estimate.

### Synthesis methods

The quantitative assessment of the included studies was performed by meta-analysis using the Review Manager (RevMan) software (Cochrane Review Manager, version 5.3). Each outcome (VO_2_max/peak, and distance walked) was expressed as mean differences (MDs) and 95% confidence interval (CI) between individuals with and without type 2 diabetes. The results were pooled using a random-effects model.

Statistical heterogeneity was assessed by the Cochran's Q test, at 0.1 significance level, and inconsistency I^2^ test. Considerable heterogeneity was indicated when I^2^ value was > 75%, according to the Cochrane Handbook for Systematic Reviews of Interventions (17). Heterogeneity among studies was investigated based on two strategies: (1) the meta-analysis was re-run by removing each study to check if one specific study explained the heterogeneity and (2) stepwise meta-regression analyses were conducted. Univariate meta-regression models were performed in STATA software (version 20) to assess clinical and methodological variables associated with CRF, i.e., BMI, age, HbA1c, and diabetes duration, based on R^2^ values and statistical significance p < 0.05. Subgroup analysis was conducted by type of ergometer (i.e., cycle ergometer and treadmill) and sex.

### Data treatment

In studies that presented the results as standard deviation (n = 31), the conversion to standard error was made by the equation SD = SEM.√sample size. The VO_2_max unit was converted from absolute (mL/min) to relative weight values (mL.kg^−1^.min^−1^) in six studies. The metabolic equivalents were converted into relative weight values (mL.kg−1.min−1) in three studies, based on the standard equation (*VO*_2_ = *METS* × 3,5) (20).

The data were combined in an unique group in studies with more than one group of individuals with and without type 2 diabetes (e.g., men and women), as suggested by the Cochrane's handbook.

## RESULTS

### Study selection

**Figure 1:**
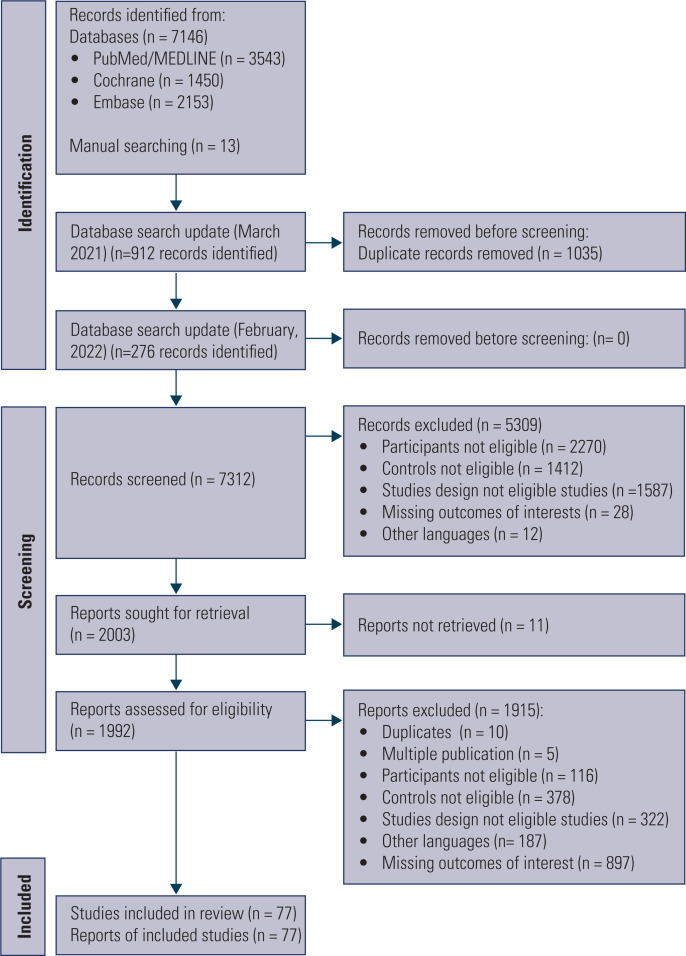
Flow diagram of included studies.

In total, 77 out of 8,347 studies identified in the data search (databases 7,146 + manual searching 13 + update 1,188) met the eligibility criteria and were included in our review. Figure 1 shows the flowchart of inclusion and exclusion criteria of studies. Meta-analysis for the VO_2_max and distance walked in the 6MWT included 72 and 5 studies, respectively.

### Study characteristics

The included studies were published from 1984 to 2022 and the sample sizes ranged from 10 (21,22) to 3,770 participants (23). A total of 8,725 individuals were included in the meta-analysis, 2,007 in the diabetes group and 6,718 in the group without diabetes. The participants were aged < 60 years in 89% of the studies. Twenty-two studies included only men, eight studies included only women, and 42 studies included both men and women. The baseline HbA1c ranged from 5.8% to 12.2% in individuals with diabetes (data available in 64 studies) and the duration of the disease ranged from 2.5 to 12.5 years (data available in 54 studies). Most of the included studies (n = 43) presented matched groups by age, sex and/or BMI. Tables 1 and 2 show the characteristics of the studies included in the VO_2_max and distance walked meta-analyses, respectively.

**Table 1:** Characteristics of the maximal cardiopulmonary test studies included in the VO_2_ meta-analysis (n = 70)

Study	Sample size (n)		Sex	Diabetes duration (years)	Matched groups	Medication	Ergometer	Protocol	VO_2_ peak/max
	DM	C							
Andrade-Mayorga and cols. (2020) (79)	13	32	M/W	NR	NR	NR	Cycle ergometer	Modified Astrand	VO_2_ peak
Baldi and cols. (2003) (24)	11	12	M/W	5.4 ± 3.1	Age, BMI, and habitual physical activity	Antidiabetics and antihypertensives	Cycle ergometer	Initial workloads 25 or 50 Wt, increments 15-25 W	VO_2_ max
Baldi and cols. (2006) (80)	13	15	M/W	5.4 ± 3.1	Age and BMI	Antidiabetics and antihypertensives	Cycle ergometer	Initial workloads 25 or 50 Wt, increments 15-25 W	VO_2_ max
Bauer and cols. (2007) (60)	11	11	M/W	NR	No	NR	Cycle ergometer	Incremental 10-20 Wt/min	VO_2_ peak
Baynard and cols. (2005) (50)	9	6	W	NR	Age	Antidiabetics	Treadmill	Starting at 2.5 mph, increased 2%mph 3.5mph reached	VO_2_ peak
Bergman and cols. (2015) (31)	15	14	M/W	NR	No	Antidiabetics	Cycle ergometer	Workload adjusted to maintain determined intensity	VO_2_ max
Boon and cols. (2007) (32)	10	10	M	7.0 ± 3.1	Weight	Antidiabetics	Cycle ergometer	Workload at 0.75 to 1.5 W.KgFFM^−1^, cadence 60 rpm	VO_2_ max
Borghouts and cols. (2002) (12)	8	8	M	NR	Weight and body composition	Antidiabetics	Cycle ergometer	Workload at 0.75 to 1.5 W.KgFFM^−1^, cadence 60 rpm	VO_2_ max
Brandenburg and cols. (1999) (33)	8	19	W	3.0 ± 2.0	Age and activity levels	NR	Cycle ergometer	Workload increases 10Wt/min.	VO_2_ max
Chance and cols. (2008) (81)	69	45	M/W	7.8 ± 5.8	Age	Antidiabetics and insulin	Cycle ergometer	Incremental 20-30 Wt/3 min.	VO_2_ peak
Colberg and cols. (2005) (35)	9	10	M/W	NR	No	NR	Cycle ergometer	Initial workload 0 Wt or 20 Wt, incremental 20 Wt/3 min., cadence of 50 rpm	VO_2_ peak
Colberg and cols. (2006) (34)	10	9	M/W	NR	No	NR	Cycle ergometer	Initial workload 0 Wt or 20 Wt, incremental 20 Wt/3 min., cadence of 50 rpm	VO_2_ peak
Cusi and cols. (2001) (25)	8	6	M/W	NR	No	Antidiabetics	Cycle ergometer	NR	VO_2_ max
Dela and cols. (1999) (21)	4	6	NR	NR	No	Antidiabetics	Cycle ergometer	NR	VO_2_ max
Devlin and cols. (1987) (26)	5	12	M	NR	No	Antidiabetics	Cycle ergometer	NR	VO_2_ max
Durrer and cols. (2017) (82)	10	9	M/W	NR	Age	Antidiabetics	Cycle ergometer	Ramp protocol (15 Wt/min at 50 rpm)	VO_2_ peak
Fluckey and cols. (1994) (83)	10	3	M/W	2.8 ± NR	Age	NR	Treadmill	Modified Naughton-Balke	VO_2_ max
Fujii and cols. (2017) (84)	12	12	M	7.5 ± 4.4	No	Antidiabetics, insulin, statins, and antihypertensives	Cycle ergometer	NR	VO_2_ peak
Green and cols. (2003) (27)	15	16	NR	NR	No	Antidiabetics, insulin, statins, and antihypertensives	Cycle ergometer	Initial workload 20 and 60 Wt, increased 20-25 Wt increments each 3 min	VO_2_ peak
Groen and cols. (2019) (85)	9	8	M/W	8 ± 3	No	Antidiabetics	Cycle ergometer	Initial workload 50-100 Wt, increases 25 Wt each 1min	VO_2_ peak
Gulsin and cols. (2020) (86)	87	36	M/W	4.7 ± 3.8	No	Antidiabetics, insulin, and antihypertensives	Cycle ergometer	Incremental	VO_2_ peak
Hansen and cols. (2014) (28)	33	18	M	5.2 ± 4.4	No	Antidiabetics, insulin, statins, and antihypertensives	Cycle ergometer	Initial workload 45 Wt, increments 45 Wt each 3-min.	VO_2_ peak
Hernández-Alvarez and cols. (2010) (9)	12	7	M/W	2.5 ± 1.9	Age and BMI	Antidiabetics and insulin	Treadmill	Stepwise	VO_2_ max
Holton and cols. (2003) (36)	9	10	M/W	NR	Age, gender, and BMI	NR	Cycle ergometer	Initial workload 0 Wt or 20 Wt, 20 Wt increment every 3 min., cadence 50 rpm.	VO_2_ peak
Huebschmann and cols. (2009) (37)	13	26	W	3.9 ± 3.9	No	Antidiabetics	Cycle ergometer	Initial workload 0 Wt, increments 10 Wt/min., cadence at 60 rpm	VO_2_ peak
Iborra and cols. (2008) (29)	14	12	M/W	9.0 ± 4.0	No	Antidiabetics, insulin, and antihypertensives	Cycle ergometer	Workload increased 10-15 Wt/min.	VO_2_ peak
Jae and cols. (2016) (23)	170	3600	M	NR	No	NR	Treadmill	Bruce	VO_2_ peak
Karavelioglu and cols. (2013) (87)	67	68	M/W	5.2 ± 4.1	Age and gender	Antidiabetics and insulin	Treadmill	Bruce	VO_2_ peak
Kasumov and cols. (2015) (38)	10	14	M/W	NR	No	Insulin	Treadmill	Incremental	VO_2_ max
Kennedy and cols. (1999) (22)	5	5	M/W	NR	No	Antidiabetics	Cycle ergometer	Incremental (2-min. stages)	VO_2_ max
Lalande and cols. (2008) (39)	8	11	M	5.0 ± 8.4	Weight and habitual activity level	Antidiabetics	Cycle ergometer	Initial workload 40 Wt, increases 15 Wt/min.	VO_2_ max
Larsen and cols. (2009) (51)	8	15	M	4.0 ± 2.8	Age and BMI	Antidiabetics	Cycle ergometer	NR	VO_2_ max
Mac Ananey and cols. (2011) (40)	9	20	W	1-5years	No	Antidiabetics, insulin, statins, and antihypertensives	Cycle ergometer	Initial workload 40 Wt, increases 20 Wt each 3 min., cadence 60 rpm	VO_2_ peak
Madsen and cols. (2015) (41)	10	13	M/W	NR	Age, height, and weight	Antidiabetics, statins, and antihypertensives	Cycle ergometer	Initial workload 80-100 Wt, increases 15 W/min., cadence 60 rpm.	VO_2_ max
Martin and cols. (1995) (10)	8	7	M	4.7 ± 3.6	Age and weight	Antidiabetics	Cycle ergometer	NR	VO_2_ max
Meex and cols. (2010) (42)	18	20	M	3.9 ± 16.5	Age, weight, and BMI	Antidiabetics	Cycle ergometer	Constant cadence 80 ± 5 rpm. At Wmax previously estimated.	VO_2_ max
Meneilly and cols. (1996) (88)	33	25	M/W	3.0 ± 5.7	Age and weight	Antidiabetics and antihypertensives	Cycle ergometer	Workload increases 16.6 Wt each 30s.	VO_2_ max
Meneilly and cols. (1999) (89)	34	19	M/W	3.0 ± 4.3	Age and weight	Antidiabetics and antihypertensives	Cycle ergometer	Workload increases 16.6 Wt each 30s.	VO_2_ max
Mogensen and cols. (2009) (52)	12	11	M	3.9 ± 3.1	Age and weight	Antidiabetics, statins, and antihypertensives	Cycle ergometer	Initial workload 60-70% HRmax, increases 30 Wt each 3 min., cadence of 60 rpm	VO_2_ max
Oberbach and cols. (2006) (67)	10	15	M/W	NR	Age and BMI	NR	Cycle ergometer	Graded	VO_2_ max
O'Connor and cols. (2012) (54)	32	32	M/W	5.1 ± 2.6	Age and BMI	Antidiabetics	Cycle ergometer	Initial workload 40 Wt, increases 30 Wt each 3 min., cadence 60 rpm	VO_2_ peak
O'Connor and cols. (2015) (53)	33	21	M	3.9 ± 2.5	Age	Antidiabetics	Cycle ergometer	Incremental	VO_2_ peak
Pinna and cols. (2021) (90)	13	13	M/W	At least 1 year	Age and sex	Antidiabetics	Cycle ergometer	Incremental	VO_2_ max
Regensteiner and cols. (1995) (55)	10	10	M/W	6.7 ± 6.8	Age, gender, weight, and physical activity	Antidiabetics	Treadmill	Modified Naughton	VO_2_ max
Regensteiner and cols. (1998) (56)	10	20	W	3.0 ± 2.0	Age, gender, weight, and physical activity	Antidiabetics	Cycle ergometer	Workload increases 10 Wt/min.	VO_2_ max
Regensteiner and cols. (2009) (57)	10	10	W	3.6 ± 0	No	Antidiabetics	Cycle ergometer	Workload increases 10 Wt/min.	VO_2_ peak
Regensteiner and cols. (2015) (43)	29	34	M/W	3.1 ± 2.8	No	Antidiabetics and statins	Cycle ergometer	Workload increases 10-25 Wt/min.	VO_2_ peak
Ribeiro and cols. (2008) (11)	21	11	M/W	8.6 ± 8.2	No	Antidiabetics, statins, and antihypertensives	Cycle ergometer	Workload increases 10-15 Wt/min.	VO_2_ max
Scalzo and cols. (2018) (44)	31	21	M/W	NR	BMI	Antidiabetics	Cycle ergometer	Workload increases 10-20 Wt/min.	VO_2_ peak
Scalzo and cols. (2022) (49)	19	22	M/W	NR	No	Antidiabetics	Cycle ergometer	Incremental	VO_2_ peak
Scheede-Bergdahl and cols. (2009) (91)	12	9	M	5.1 ± 3.8	Age, weight, and body fat	Antidiabetics, statins, and antihypertensives	Cycle ergometer	NR	VO_2_ peak
Scheede-Bergdahl and cols. (2014) (45)	12	9	M	5.1 ± 3.8	No	Antidiabetics, statins and antihypertensives	Cycle ergometer	NR	VO_2_ peak
Schneider and cols. (1984) (46)	20	11	M	NR	Age, gender, and weight	None	Cycle ergometer	Workload increments of 25 Wt, each 3 min.	VO_2_ max
Schneider and cols. (1988) (30)	16	9	M/W	NR	Age, gender, and weight	None	Cycle ergometer	NR	VO_2_ max
Schreuder and cols. (2014) (68)	27	9	M	NR	Age, gender, and weight	Antidiabetics, insulin, statins, and antihypertensives	Cycle ergometer	Initial workload 10 Wt, increases 10 Wt/min., cadence 60-80 rpm	VO_2_ max
Segerstrom and cols. (2011) (69)	39	53	M	NR	Age	Antidiabetics and insulin	Cycle ergometer	Initial workload 30 Wt, increases 15 Wt/min., cadence 60 rpm	VO_2_ peak
Simões and cols. (2010) (13)	10	10	NR	NR	No	Antidiabetics	Cycle ergometer	Initial workload 15 Wt, increases 15 Wt each 3 min.	VO_2_ max
Simões and cols. (2013) (47)	10	10	M/W	6.0 ±1.1	Age, weight, and BMI	Antidiabetics and antihypertensives	Cycle ergometer	Initial workload 15 Wt, increases 15 Wt each 3 min.	VO_2_ peak
Suk and cols. (2015) (92)	12	12	W	7.8 ± 2.1	BMI	None	Cycle ergometer	Initial workload 20% of Wmax, increments 30-60% Wmax each 3 min.	VO_2_ max
Tadic and cols. (2021) (65)	30	55	M/W	NR	No	Antidiabetics and insulin	Treadmill	Modified Bruce	VO_2_ peak
Tobin and cols. (2008) (93)	8	7	M	4.9 ± 3.3	Age, gender, and BMI	Antidiabetics	Cycle ergometer	Graded	VO_2_ max
Van Tienen and cols. (2012) (61)	8	12	M	12.5 ± 7.7	Age, weight, and BMI	NR	Cycle ergometer	Incremental	VO_2_ peak
Vind and cols. (2011) (94)	13	13	M	3.7 ± 2.8	Age and BMI	Antidiabetics and antihypertensives	Cycle ergometer	Initial workload 60-70% HRmax, increases 30 Wt each 3 min., cadence of 60 rpm	VO_2_ peak
Vukomanovic and cols. (2020) (95)	64	72	M/W	NR	No	NR	Treadmill	Modified Bruce	VO_2_ peak
Vukomanovic and cols. (2019) (96)	70	80	M/W	3 (1-5)	No	Antidiabetics and insulin	Treadmill	Modified Bruce	VO_2_ peak
Vukomanovic and cols. (2019) (97)	53	62	M/W	NR	No	Antidiabetics	Treadmill	Modified Bruce	VO_2_ peak
Wilkerson and cols. (2011) (48)	12	12	M	<5 years	Age and weight	Antidiabetics and antihypertensives	Cycle ergometer	Ramp (15 Wt/min.)	VO_2_ max
Wilmot and cols. (2014) (14)	20	20	M/W	4.7 ± 4.0	Age	Antidiabetics and antihypertensives	Cycle ergometer	NR	VO_2_ max
Wilson and cols. (2017) (58)	17	16	M/W	8.3 ± 9.5	Age, gender, BMI, physical activity	Antidiabetics and insulin	Cycle ergometer	Initial workload 25-50 Wt, increases 25-50 Wt each 1min	VO_2_ peak
Yu and cols. (2016) (70)	180	1594	NR	NR	Age and gender	NR	Treadmill	Bruce	VO_2_ peak
Zbinden-Foncea and cols. (2013) (63)	10	5	NR	NR	No	Antidiabetics	Cycle ergometer	Incremental	VO_2_ max
Zierath and cols. (1996) (72)	7	7	M	5 ± 5.2	Age and BMI	Antidiabetics	Cycle ergometer	Initial workload 50 Wt, increases 50 Wt each 5 min.	VO_2_ max

Data are presented in mean ± SD or (range); DM: diabetes; C: control; W: women; M: men; NR: not reported; Wt: watts; min.: minutes; mph: miles per hour; KgFFM: kilogram of free fat mass; rpm: rotations per minute; Wmax: maximal workload; HRmax: maximal heart rate; Tmax.: maximal exercise test duration; VO_2_ peak: peak oxygen consumption; VO_2_max: maximal oxygen consumption.

**Table 2:** Characteristics of the six-minute walk test studies included in the meta-analysis (n = 5)

Study	Sample Size		Sex	Diabetes duration (years)	Medications	Protocol	Estim.VO_2_ (mL/kg/min)	
	DM	C					DM	C
Awotidebe and cols. (2014) (98)	35	35	M/W	NR	NR	ATS	10.2 ± 1.5	10.9 ± 1.3
Awotidebe and cols. (2016) (99)	125	125	M/W	<5 years	Antidiabetics	ATS	7.6 ± 0.6	9.6 ± 0.6
Heberle and cols. (2021) (64)	13	13	W	11.92 ± 10.77	Antidiabetics and statins	NR	NR	NR
IJzerman and cols. (2012) (59)	137	19	M/W	NR	NR	NR	NR	NR
Ozdirenc and cols. (2003) (62)	30	30	M/W	7.1 ± 6.2	Antidiabetics and insulin	NR	14.6 ± 2.9	17.0 ± 1.8

Data are presented in mean ± SD; DM: diabetes; C: control; M: men; W: women; NR: not reported; ATS: American Thoracic Society; VO_2_: maximal oxygen consumption; Estim.VO_2_ : Estimated VO_2_.

A total of 73 included studies reported the BMI. Among the type 2 diabetes group, 1.3% (n = 1), 43.8% (n = 32), and 54.8% (n = 40) were classified as normal weight, overweight and obese, whereas lean and obese individuals with diabetes were pooled to be analyzed in four studies. We observed a high prevalence of patients classified as overweight (59.2%, n = 40) and obese (22.2%, n = 17) in the group without diabetes.

A total of 46 out of the 77 included studies reported habitual physical activity (PA). Eight studies reported that participants did not participate in regular exercise programs (21,24–30), 22 studies reported that participants were sedentary or physically inactive (9,11,12,31-49), 10 studies reported similar PA level in groups with and without diabetes (50–59), two studies reported that habitual PA scores were higher in the diabetes group (60,61), three studies showed higher levels of PA in the group without diabetes (13,62,63) and one study reported that individuals practiced physical exercises for more than six months (64).

We classified all 77 included studies as poor quality and they achieved a mean score of 2.4/8 in the modified NOS (Supplementary File 2). No study has reached the maximum score (3 points) in the selection domain, although seven studies scored 2 points and 46 studies scored 1 point. Eight studies reached maximal score (2 points) and 33 scored 1 point in the comparability domain. Finally, 22 studies reached the maximal score (2 points) and 35 studies scored 1 point in the outcome domain. Mac Ananey and cols. (40) reached the highest score (5 points), whereas four studies scored zero points (9,21,25,65).

We evaluated the publication bias using a funnel plot for the VO_2_max (Supplementary File 3). The points for the missing studies would be on the bottom of the plot. Since most of this area contains regions of small sample size, publication bias is unlikely to be the cause of this asymmetry. The analyzed studies did not run further tests to distinguish chance from real asymmetry.

### Results of syntheses

**Figure 2:**
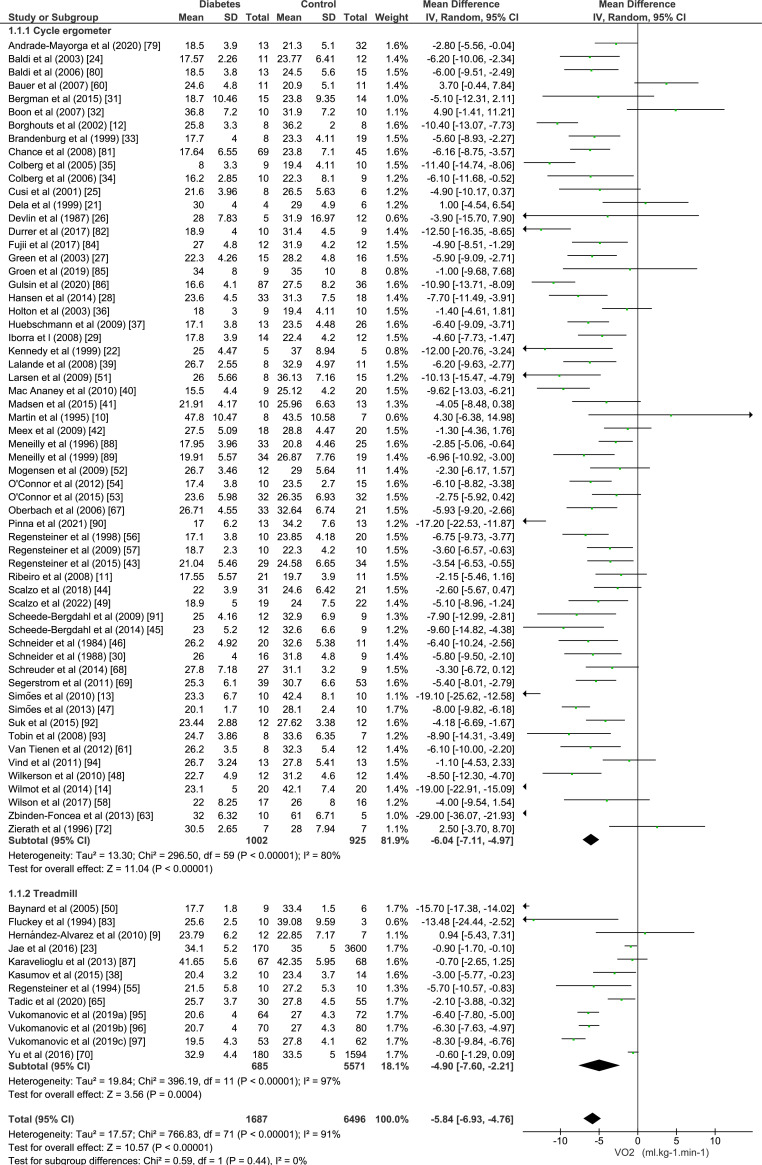
Forest plot of the maximal oxygen consumption (VO_2_max) evaluated in maximal cardiopulmonary exercise tests.

Figure 2 shows the data about the meta-analysis of VO_2_max, which shows that individuals with diabetes had lower VO_2_max/peak [−5.84 mL.kg^−1^.min^−1^ (95% CI −6.93, −4.76 mL.kg^−1^.min^−1^, p = <0.0001); I^2^ = 91%, p for heterogeneity < 0.0001] compared to the group without diabetes. We included 8,183 individuals from 72 studies in this analysis. Most studies used cycle ergometer (n = 44) and 26 studies reported VO_2_max.

Absolute changes in VO_2_max in studies conducted with type 2 diabetes patients compared with group without type 2 diabetes. Squares represent study-specific estimates; diamonds represent pooled estimates of random-effects meta-analyses

Heterogeneity in VO_2_max analyses was classified as high (I^2^ = 91%). We did not observe substantial change in heterogeneity at each study removal. Subgroup analyses (Supplementary File 4) showed that heterogeneity remained unchanged when studies were exclusively conducted with men (I^2^ = 82.6%; p < 0.001) or women (I^2^ = 93.9%; p < 0.001).

Meta-regression analyses of studies included in VO_2_max analyses indicated that BMI partly explained the heterogeneity among studies [adjusted R^2^ = 10.75%; coefficient −0.4988; 95%CI (−0.94; −0.05); p=0.03]. Age (adjusted R^2^ = −2.10%; p = 0.99), HbA1c (adjusted R^2^ = 4.48%; p = 0.08), and diabetes duration (adjusted R^2^ = −4.21%; p = 0.69) were not associated with differences among studies (Supplementary File 5).

**Figure 3:**
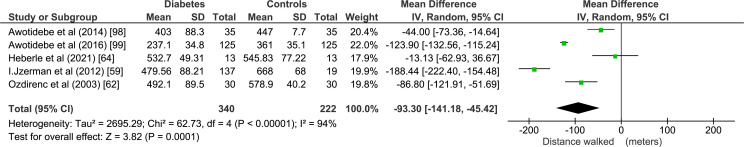
Forest plot of the distance walked in the six minute walk test (6MWT).

We included five studies in the meta-analysis of the distance walked evaluated by 6MWT. Subjects with diabetes walked −93.30 meters (95% CI −141.2, −45.4 meters, p > 0.0001; I^2^ = 94%, p for heterogeneity < 0.0001) compared to the group without diabetes (Figure 3).

Absolute changes in the distance walked in the 6MWT in studies conducted with type 2 diabetes patients compared with control group. Squares represent study-specific estimates; diamonds represent pooled estimates of random-effects meta-analyses

## DISCUSSION

To our knowledge, this systematic review with meta-analysis was the first study comparing CRF between individuals with and without diabetes, in which we observed that individuals with type 2 diabetes presented lower CRF evaluated by VO_2_max. This is essential because VO_2_max is a measure associated with health and this review included studies with different designs to broadly analyze this variable in diabetes and non diabetes groups. The lower VO_2_max values indicated may be useful to qualify future studies about physical rehabilitation and physical activity for individuals with type 2 diabetes.

Cardiac, respiratory, and skeletal muscular systems determine VO_2_max (66). This assumption is supported by studies that indicate that VO_2_max reduction is associated with diastolic dysfunction and/or impaired myocardium perfusion during exercise (57), as well as with abnormalities in skeletal muscle morphology (67), VO_2_ kinetics (O_2_ uptake/use) (54,56), endothelial dysfunction (43,68,69), blood viscosity (55), and glycemic profile/control (69). Changes in these components and in their integration can lead to VO_2_max impairment. Our study only assessed glycemic control based on HbA1c. However, meta-regression results did not associate HbA1c with VO_2_max in patients with diabetes.

We observed lower values of VO_2_max in individuals with diabetes compared with the other group, however, 22 out of the 70 included studies did not show differences between the groups. This could be explained by: 1. One study had a diabetes group with higher levels of physical activity than the group without diabetes (61); 2. Another study included only individuals with obesity in the group without diabetes with VO_2_max values lower than predicted for normal weight individuals (25); 3. One study included a 10 times smaller sample size in the diabetes group than in the group without diabetes (70); and 4. Four studies did not control for comorbidities, such as cardiovascular, endocrine or renal diseases, in groups with and without diabetes (14,25,71,72).

The 6MWT has been used to estimate exercise capacity in adults with diabetes. However, results have shown moderate correlation between estimated VO_2_max and 6MWT (73), which suggest that 6MWT can be used to assess the patients’ ability to maintain the exercise, but not to estimate VO_2_max. We observed a mean reduction of 93 meters in the distance walked among individuals with diabetes compared to the group without diabetes. Studies showed that reductions of 25-30 meters in distance walked in patients with coronary artery diseases and pulmonary diseases were associated with increased risk of death (74,75). However, the minimal clinically significant difference values of distance walked are not established among patients with diabetes. Although our study found significant reduction in distance walked evaluated by 6MWT in patients with diabetes, our results have limited generalizability due to few included studies.

The first strategy adopted to explore heterogeneity was to remove each study from the analyses, which did not cause changes. Despite the group without diabetes being restricted to individuals without diabetes, we cannot assert their health status. Moreover, factors such as obesity and/or age could explain similar VO_2_max between the diabetes group and the group without diabetes in one third of the analyzed studies.

The high heterogeneity in the VO_2_max meta-analysis was explored by sensitive analyses considering the ergometer and sex and also by performing a meta-regression analysis. The type of ergometer used in the tests did not change the results of the meta-analysis, as well as the subgroup analyses based on sex. Besides, meta-regression analysis applied to VO_2_max showed that age, HbA1c and diabetes duration could not explain the high heterogeneity presented in VO_2_max meta-analysis, but BMI partly explained it. We believe that the high I^2^ in the VO_2_max analysis is related to different magnitudes of these effects in the different studies shown. However, most of them present the same direction of effects. Therefore, the practical/clinical implications are that despite the high heterogeneity, the direction of effect shows lower cardiorespiratory fitness in individuals with diabetes, but we cannot estimate the exact magnitude of the difference between type 2 diabetes and control groups.

Higher levels of CRF may coexist with higher BMI (76). Hemmingsson and cols. observed a reduction of the normal weight and high CRF category (relative change −30%) when analyzing time trends combinations between CRF and BMI (1995-2020), an increase in overweight and low CRF (relative change +34%) and in obesity and low CRF (relative change +154%) categories. Studies show that the risk of cardiovascular disease and all-cause mortality in individuals with obesity varied by CRF (77), such as among individuals with diabetes (78).

Our study has some limitations. Although the search was not limited by language, the included studies were only in Portuguese, English, and Spanish. It was a challenge to summarize the results of this review, since different protocols were used to evaluate CRF. Moreover, there was a considerable variation among the studies about pharmacological treatments that diabetes patients received, some studies did not mention the drugs used to treat health conditions other than diabetes, and most studies did not mention the drug doses used. Most of the included studies were carried out in participants with a mean age of less than 60 years, and the highest prevalence of type 2 diabetes is found in older adults. Another challenge was the wide range of duration of diabetes, because the CRF can change along with diabetes duration. Therefore, these are limitations that could affect the generalization of our outcomes. Additionally, the overall quality of the studies was low, indicating increased risk of bias in many of them, however, there are fewer instruments to evaluate risk of bias of observational studies and they are less accurate compared to those evaluating clinical trials.

The strength of this systematic review is that we could summarize how lower the VO_2_max is reduced in individuals with type 2 diabetes compared with the group without type 2 diabetes, due to many included studies (n = 77). Furthermore, the exploratory analyses to explore the high heterogeneity followed all guidelines for systematic reviews.

In conclusion, individuals with type 2 diabetes showed lower CRF than the group without diabetes. CRF was evaluated by the VO_2_max individuals attained in maximal cardiopulmonary exercise testing, and was partially influenced by the BMI, but not influenced by age or sex of this population. Moreover, lower distance walked was observed in the group with diabetes. This review emphasizes exercise as a component to treat and to control diabetes that should be evaluated and prescribed individually.

## Data Availability

data are available on request from the authors.

## Supplemental file 1 Literature search strategy

**Table:**

PubMed
#1(“Diabetes Mellitus, Type 2”[title] OR “Diabetes Mellitus”[title] OR “Diabetes Mellitus, Noninsulin-Dependent”[title] OR “Diabetes Mellitus, Ketosis-Resistant”[title] OR “Diabetes Mellitus, Ketosis Resistant”[title] OR “Ketosis-Resistant Diabetes Mellitus”[title] OR “Diabetes Mellitus, Non Insulin Dependent”[title] OR “Diabetes Mellitus, Non-Insulin-Dependent”[title] OR “Non-Insulin-Dependent Diabetes Mellitus”[title] OR “Diabetes Mellitus, Stable”[title] OR “Stable Diabetes Mellitus”[title] OR “Diabetes Mellitus, Type II”[title] OR “NIDDM”[title] OR “Diabetes Mellitus, Noninsulin Dependent”[title] OR “Diabetes Mellitus, Maturity-Onset”[title] OR “Diabetes Mellitus, Maturity Onset”[title] OR “Maturity-Onset Diabetes Mellitus”[title] OR “Maturity Onset Diabetes Mellitus”[title] OR “MODY”[title] OR “Diabetes Mellitus, Slow-Onset”[title] OR “Diabetes Mellitus, Slow Onset”[title] OR “Slow-Onset Diabetes Mellitus”[title] OR “Type 2 Diabetes Mellitus”[title] OR “Noninsulin-Dependent Diabetes Mellitus”[title] OR “Noninsulin Dependent Diabetes Mellitus”[title] OR “Maturity-Onset Diabetes”[title] OR “Diabetes, Maturity-Onset”[title] OR “Maturity Onset Diabetes”[title] OR “Type 2 Diabetes”[title] OR “Diabetes, Type 2”[title] OR “Diabetes Mellitus, Adult-Onset”[title] OR “Adult-Onset Diabetes Mellitus”[title] OR “Diabetes Mellitus, Adult Onset”[title] OR “DM2”[title] OR “diabetics”[tiab])
#2(“Exercise Therapy”[Mesh] OR “Resistance Training”[Mesh] OR “Muscle Stretching Exercises”[Mesh] OR “Exercise Movement Techniques”[Mesh] OR “Exercise”[Mesh] OR “Cardiorespiratory Fitness”[Mesh] OR “evaluation cardiopulmonary” OR “Ergospirometry” OR “Functional capacity” OR “Tests of exercise endurance” OR “six-minute walk” OR “Exercise testing” OR “stress testing” OR “Oxygen Consumption” OR “Cardiopulmonary exercise testing” OR “Exercises” OR “Physical Exercise” OR “Physical Exercise” OR “Physical Exercises” OR “Isometric Exercises” OR “Isometric Exercise” OR “Warm Up Exercise” OR “Aerobic Exercises” OR “Aerobic Exercise” OR “Exercise Therapies” OR “Pilates Training” OR “Strength Training” OR “Strengthening Programs” OR “Weight Lifting Exercise Program” OR “Weight Bearing Strengthening Program” OR “Weight Bearing Exercise Program” OR “Effort test” OR “Fitness, Cardiorespiratory” OR “peak oxygen uptake” OR “maximal oxygen consumption” OR “peak oxygen consumption”)
#1 AND #2
**Embase**
#1 ‘non insulin dependent diabetes mellitus’/exp
#2‘exercise’/exp OR ‘exercise tests’/exp OR ‘cardiorespiratory fistness’/exp OR cardiopulmonary exercise test’/exp OR ‘ergoespirometry’/exp OR ‘six-minute walk test’/exp OR ‘maximal oxygen uptake’/exp OR functional status assessment’/exp or ‘treadmill exercise test’/exp
#3‘non diabetic patient’/exp OR ‘control group’/exp OR ‘normal human’/exp
#1 AND #2 AND #3
**Cochrane**
“exercise”
AND
“diabetes mellitus, type 2”

## Supplemental file 2 Risk of bias by Newcastle-Ottawa Scale (n = 77 studies)

**Table:**

Study	Year	Selection	Comparability	Outcome	Total score
Andrade-Mayorga and cols. (79)	2020	1	0	2	3
Awotidebe and cols. (98)	2014	1	1	1	3
Awotidebe and cols. (99)	2016	2	0	1	3
Baldi and cols. (24)	2003	0	0	1	1
Baldi and cols. (80)	2006	1	0	0	1
Bauer and cols. (60)	2007	1	0	1	2
Baynard and cols. (50)	2005	1	0	1	2
Bergman and cols. (31)	2015	0	0	1	1
Boon and cols. (32)	2007	1	0	1	2
Borghouts and cols. (12)	2002	0	0	1	1
Brandenburg and cols. (33)	1999	2	2	0	4
Chance and cols. (81)	2008	0	1	0	1
Colberg and cols. (35)	2005	0	0	1	1
Colberg and cols. (34)	2006	0	0	1	1
Cusi and cols. (25)	2001	0	0	0	0
Dela and cols. (21)	1999	0	0	0	0
Devlin and cols. (26)	1987	0	1	0	1
Durrer and cols. (82)	2017	2	0	2	4
Fluckey and cols. (83)	1994	1	1	0	2
Fujii and cols. (84)	2017	0	0	2	2
Green and cols. (27)	2003	2	0	1	3
Groen and cols. (85)	2019	1	0	2	3
Gulsin and cols. (86)	2020	1	0	1	1
Hansen and cols. (28)	2014	0	0	2	2
Heberle and cols. (64)	2021	1	1	1	3
Hernández-Alvarez and cols. (9)	2010	0	0	0	0
Holton and cols. (36)	2003	1	1	1	3
Huebschmann (37)	2009	1	0	1	2
Iborra and cols. (29)	2008	1	1	1	3
IJzerman and cols. (59)	2012	1	1	1	3
Jae and cols. (23)	2016	1	0	1	2
Karavelioglu and cols. (87)	2013	1	0	1	2
Kasumov and cols. (38)	2015	1	0	0	1
Kennedy and cols. (22)	1999	1	0	1	2
Lalande and cols. (39)	2008	0	0	1	1
Larsen and cols. (51)	2009	0	2	0	2
Mac Ananey and cols. (40)	2011	2	2	1	5
Madsen and cols. (41)	2015	0	2	1	3
Martin and cols. (10)	1995	0	2	0	2
Meex and cols. (42)	2010	1	2	0	3
Meneilly and cols. (88)	1996	1	1	1	3
Meneilly and cols. (89)	1999	0	1	1	2
Mogensen and cols. (52)	2009	2	1	1	4
Oberbach and cols. (67)	2006	1	1	1	3
O'Connor and cols. (54)	2012	0	1	2	3
O'Connor and cols. (53)	2015	1	1	2	4
Ozdirenc and cols. (62)	2003	1	1	2	4
Pinna and cols. (90)	2021	1	1	2	4
Regensteiner and cols. (55)	1995	1	2	0	3
Regensteiner and cols. (56)	1998	1	1	2	4
Regensteiner and cols. (57)	2009	1	1	2	4
Regensteiner and cols. (43)	2015	1	1	2	4
Ribeiro and cols. (11)	2008	1	1	1	3
Scalzo and cols. (44)	2018	1	1	1	3
Scalzo and cols. (49)	2022	2	0	1	3
Scheede-Bergdahl and cols. (91)	2009	1	1	1	3
Scheede-Bergdahl and cols. (45)	2014	1	1	1	3
Schneider and cols. (46)	1984	1	2	0	3
Schneider and cols. (30)	1988	1	1	2	4
Schreuder and cols. (68)	2014	0	1	2	3
Segerstrom and cols. (69)	2011	1	1	2	4
Simões and cols. (13)	2010	0	0	2	2
Simões and cols. (47)	2013	1	0	2	3
Suk and cols. (92)	2015	1	1	2	4
Tadic and cols. (65)	2021	0	0	0	0
Tobin and cols. (93)	2008	0	1	2	3
Van Tienen and cols. (61)	2012	1	1	1	3
Vind and cols. (94)	2011	1	1	2	4
Vukomanovic and cols. (95)	2020	1	1	0	2
Vukomanovic and cols. (96)	2019	1	0	0	1
Vukomanovic and cols. (97)	2019	1	0	0	1
Wilkerson and cols. (48)	2011	1	1	0	2
Wilmot and cols. (14)	2014	1	0	1	2
Wilson and cols. (58)	2017	0	1	0	1
Yu and cols. (70)	2016	1	0	2	3
Zbinden-Foncea and cols. (63)	2013	1	0	1	2
Zierath and cols. (72)	1996	0	0	2	2

Domain selection checked: the representativeness of the sample, sample size and diagnosis of type 2 diabetes (maximum score: 3 points); domain comparability checked: confounding factors, i.e. if groups (diabetes and controls) were matched by body mass index (BMI), age and/or sex (maximum score: were 2 points); domain outcome checked: the blinded assessment and statistical tests employed (maximum score: 2 points). The maximum score was eight and the classification of the studies were: (1) good quality: 2-3 points in the selection domain, 1-2 points in the comparability domain and 2-3 points in the outcome domain; (2) fair quality: 1 point in the selection domain, 1-2 points in the comparability domain and 1-2 points in the outcome domain; and (3) poor quality: 0 points in any domains.

## Supplemental file 4 Subgroup analyses

**Table:**

	N. of studies	VO_2_			
		MD	95% CI	p	I^2^
Overall results	72	−5.84	(−6.93; −4.76)	<0.001	91.0%
Men	22	−4.84	(−6.60; −3.08)	<0.001	82.6%
Women	7	−7.45	(−11.52; −3.39)	<0.001	93.9%

MD: mean difference; CI: confidence interval; VO_2_: maximal oxygen consumption; I^2^: inconsistency I^2^ test.

## Supplemental file 5 Meta-regression analyses

**Table:**

Covariates	N of obs.	Coefficient	95%IC	p	Adjusted R^2^
BMI	68	−0.4988	−0.94; −0.05	0.03	10.75%
Age	72	0.0005	−0.13; 0.13	0.99	−2.10%
HbA1c	62	−0.7480	−1.58; 0.08	0.08	4.48%
Duration of diabetes	40	0.1221	−0.49; 0.73	0.69	−4.21%

BMI: body mass index; HbA1c: glycated haemoglobin.

## References

[1] Kaze AD, Agoons DD, Santhanam P, Erqou S, Ahima RS, Echouffo-Tcheugui JB (2022). Correlates of cardiorespiratory fitness among overweight or obese individuals with type 2 diabetes. BMJ Open Diabetes Res Care.

[2] Zafrir B, Azaiza M, Gaspar T, Dobrecky-Mery I, Azencot M, Lewis BS (2015). Low cardiorespiratory fitness and coronary artery calcification: Complementary cardiovascular risk predictors in asymptomatic type 2 diabetics. Atherosclerosis.

[3] Balducci S, Zanuso S, Cardelli P, Salvi L, Mazzitelli G, Bazuro A (2012). Changes in physical fitness predict improvements in modifiable cardiovascular risk factors independently of body weight loss in subjects with type 2 diabetes participating in the Italian Diabetes and Exercise Study (IDES). Diabetes Care.

[4] Laukkanen JA, Zaccardi F, Khan H, Kurl S, Jae SY, Rauramaa R (2016). Long-term Change in Cardiorespiratory Fitness and All-Cause Mortality: A Population-Based Follow-up Study. Mayo Clin Proc.

[5] Myers J, de Souza E, Silva CG, Doom R, Fonda H, Chan K, Kamil-Rosenberg S (2019). Cardiorespiratory Fitness and Health Care Costs in Diabetes: The Veterans Exercise Testing Study. Am J Med.

[6] American Thoracic Society; American College of Chest Physicians (2003). ATS/ACCP Statement on cardiopulmonary exercise testing. Am J Respir Crit Care Med.

[7] Ross R, Blair SN, Arena R, Church TS, Després JP, Franklin BA (2016). Importance of Assessing Cardiorespiratory Fitness in Clinical Practice: A Case for Fitness as a Clinical Vital Sign: A Scientific Statement From the American Heart Association. Circulation.

[8] Ferguson B (2014). ACSM's Guidelines for Exercise Testing and Prescription 9th Ed. 2014. J Can Chiropr Assoc.

[9] Hernández-Alvarez MI, Thabit H, Burns N, Shah S, Brema I, Hatunic M (2010). Subjects with early-onset type 2 diabetes show defective activation of the skeletal muscle PGC-1{alpha}/Mitofusin-2 regulatory pathway in response to physical activity. Diabetes Care.

[10] Martin IK, Katz A, Wahren J (1995). Splanchnic and muscle metabolism during exercise in NIDDM patients. Am J Physiol.

[11] Ribeiro IC, Iborra RT, Neves MQ, Lottenberg SA, Charf AM, Nunes VS (2008). HDL atheroprotection by aerobic exercise training in type 2 diabetes mellitus. Med Sci Sports Exerc.

[12] Borghouts LB, Wagenmakers AJ, Goyens PL, Keizer HA (2002). Substrate utilization in non-obese Type II diabetic patients at rest and during exercise. Clin Sci (Lond).

[13] Simões HG, Moreira SR, Moffatt RJ, Campbell CS (2010). Methods to identify the anaerobic threshold for type-2 diabetic and non-diabetic subjects. Arq Bras Cardiol.

[14] Wilmot EG, Leggate M, Khan JN, Yates T, Gorely T, Bodicoat DH (2014). Type 2 diabetes mellitus and obesity in young adults: the extreme phenotype with early cardiovascular dysfunction. Diabet Med.

[15] Abushamat LA, McClatchey PM, Scalzo RL, Schauer I, Huebschmann AG, Nadeau KJ (2020). Mechanistic Causes of Reduced Cardiorespiratory Fitness in Type 2 Diabetes. J Endocr Soc.

[16] Leenders M, Verdijk LB, van der Hoeven L, Adam JJ, van Kranenburg J, Nilwik R (2013). Patients with type 2 diabetes show a greater decline in muscle mass, muscle strength, and functional capacity with aging. J Am Med Dir Assoc.

[17] Higgins JPT, Thomas J, Chandler J (2019). Cochrane handbook for systematic reviews of interventions.

[18] Rethlefsen ML, Kirtley S, Waffenschmidt S, Ayala AP, Moher D, Page MJ (2021). PRISMA-S Group. PRISMA-S: an extension to the PRISMA Statement for Reporting Literature Searches in Systematic Reviews. Syst Rev.

[19] Wells GA, Shea B, O'Connell D, Peterson J, Welch V, Losos M The Newcastle-Ottawa Scale (NOS) for assessing the quality of nonrandomised studies in meta-analyses.

[20] Ainsworth BE, Haskell WL, Whitt MC, Irwin ML, Swartz AM, Strath SJ (2000). Compendium of physical activities: an update of activity codes and MET intensities. Med Sci Sports Exerc.

[21] Dela F, Mikines KJ, Larsen JJ, Galbo H (1999). Glucose clearance in aged trained skeletal muscle during maximal insulin with superimposed exercise. J Appl Physiol (1985).

[22] Kennedy JW, Hirshman MF, Gervino EV, Ocel JV, Forse RA, Hoenig SJ (1999). Acute exercise induces GLUT4 translocation in skeletal muscle of normal human subjects and subjects with type 2 diabetes. Diabetes.

[23] Jae SY, Franklin BA, Choo J, Yoon ES, Choi YH, Park WH (2016). Fitness, Body Habitus, and the Risk of Incident Type 2 Diabetes Mellitus in Korean Men. Am J Cardiol.

[24] Baldi JC, Aoina JL, Oxenham HC, Bagg W, Doughty RN (2003). Reduced exercise arteriovenous O2 difference in Type 2 diabetes. J Appl Physiol (1985).

[25] Cusi KJ, Pratipanawatr T, Koval J, Printz R, Ardehali H, Granner DK (2001). Exercise increases hexokinase II mRNA, but not activity in obesity and type 2 diabetes. Metabolism.

[26] Devlin JT, Hirshman M, Horton ED, Horton ES (1987). Enhanced peripheral and splanchnic insulin sensitivity in NIDDM men after single bout of exercise. Diabetes.

[27] Green DJ, Walsh JH, Maiorana A, Best MJ, Taylor RR, O'Driscoll JG (2003). Exercise-induced improvement in endothelial dysfunction is not mediated by changes in CV risk factors: pooled analysis of diverse patient populations. Am J Physiol Heart Circ Physiol.

[28] Hansen D, Dendale P (2014). Modifiable predictors of chronotropic incompetence in male patients with type 2 diabetes. J Cardiopulm Rehabil Prev.

[29] Iborra RT, Ribeiro IC, Neves MQ, Charf AM, Lottenberg SA, Negrão CE (2008). Aerobic exercise training improves the role of high-density lipoprotein antioxidant and reduces plasma lipid peroxidation in type 2 diabetes mellitus. Scand J Med Sci Sports.

[30] Schneider SH, Kim HC, Khachadurian AK, Ruderman NB (1988). Impaired fibrinolytic response to exercise in type II diabetes: effects of exercise and physical training. Metabolism.

[31] Bergman BC, Brozinick JT, Strauss A, Bacon S, Kerege A, Bui HH (2015). Serum sphingolipids: relationships to insulin sensitivity and changes with exercise in humans. Am J Physiol Endocrinol Metab.

[32] Boon H, Blaak EE, Saris WH, Keizer HA, Wagenmakers AJ, van Loon LJ (2007). Substrate source utilisation in long-term diagnosed type 2 diabetes patients at rest, and during exercise and subsequent recovery. Diabetologia.

[33] Brandenburg SL, Reusch JE, Bauer TA, Jeffers BW, Hiatt WR, Regensteiner JG (1999). Effects of exercise training on oxygen uptake kinetic responses in women with type 2 diabetes. Diabetes Care.

[34] Colberg SR, Parson HK, Nunnold T, Herriott MT, Vinik AI (2006). Effect of an 8-week resistance training program on cutaneous perfusion in type 2 diabetes. Microvasc Res.

[35] Colberg SR, Parson HK, Nunnold T, Holton DR, Swain DP, Vinik AI (2005). Change in cutaneous perfusion following 10 weeks of aerobic training in Type 2 diabetes. J Diabetes Complications.

[36] Holton DR, Colberg SR, Nunnold T, Parson HK, Vinik AI (2003). The effect of an aerobic exercise training program on quality of life in type 2 diabetes. Diabetes Educ.

[37] Huebschmann AG, Reis EN, Emsermann C, Dickinson LM, Reusch JE, Bauer TA (2009). Women with type 2 diabetes perceive harder effort during exercise than nondiabetic women. Appl Physiol Nutr Metab.

[38] Kasumov T, Solomon TP, Hwang C, Huang H, Haus JM, Zhang R (2015). Improved insulin sensitivity after exercise training is linked to reduced plasma C14:0 ceramide in obesity and type 2 diabetes. Obesity (Silver Spring).

[39] Lalande S, Gusso S, Hofman PL, Baldi JC (2008). Reduced leg blood flow during submaximal exercise in type 2 diabetes. Med Sci Sports Exerc.

[40] Mac Ananey O, Malone J, Warmington S, O'Shea D, Green S, Egaña M (2011). Cardiac output is not related to the slowed O2 uptake kinetics in type 2 diabetes. Med Sci Sports Exerc.

[41] Madsen SM, Thorup AC, Overgaard K, Jeppesen PB (2015). High Intensity Interval Training Improves Glycaemic Control and Pancreatic β Cell Function of Type 2 Diabetes Patients. PLoS One.

[42] Meex RC, Schrauwen-Hinderling VB, Moonen-Kornips E, Schaart G, Mensink M, Phielix E (2010). Restoration of muscle mitochondrial function and metabolic flexibility in type 2 diabetes by exercise training is paralleled by increased myocellular fat storage and improved insulin sensitivity. Diabetes.

[43] Regensteiner JG, Bauer TA, Huebschmann AG, Herlache L, Weinberger HD, Wolfel EE (2015). Sex differences in the effects of type 2 diabetes on exercise performance. Med Sci Sports Exerc.

[44] Scalzo RL, Bauer TA, Harrall K, Moreau K, Ozemek C, Herlache L (2018). Acute vitamin C improves cardiac function, not exercise capacity, in adults with type 2 diabetes. Diabetol Metab Syndr.

[45] Scheede-Bergdahl C, Bergdahl A, Schjerling P, Qvortrup K, Koskinen SO, Dela F (2014). Exercise-induced regulation of matrix metalloproteinases in the skeletal muscle of subjects with type 2 diabetes. Diab Vasc Dis Res.

[46] Schneider SH, Amorosa LF, Khachadurian AK, Ruderman NB (1984). Studies on the mechanism of improved glucose control during regular exercise in type 2 (non-insulin-dependent) diabetes. Diabetologia.

[47] Simões HG, Asano RY, Sales MM, Browne RA, Arsa G, Motta-Santos D (2013). Type 2 diabetes elicits lower nitric oxide, bradykinin concentration and kallikrein activity together with higher DesArg(9)-BK and reduced post-exercise hypotension compared to non-diabetic condition. PLoS One.

[48] Wilkerson DP, Poole DC, Jones AM, Fulford J, Mawson DM, Ball CI (2011). Older type 2 diabetic males do not exhibit abnormal pulmonary oxygen uptake and muscle oxygen utilization dynamics during submaximal cycling exercise. Am J Physiol Regul Integr Comp Physiol.

[49] Scalzo RL, Schauer IE, Rafferty D, Knaub LA, Kvaratskhelia N, Johnson TK (2022). Single-leg exercise training augments in vivo skeletal muscle oxidative flux and vascular content and function in adults with type 2 diabetes. J Physiol.

[50] Baynard T, Franklin RM, Goulopoulou S, Carhart R, Kanaley JA (2005). Effect of a single vs multiple bouts of exercise on glucose control in women with type 2 diabetes. Metabolism.

[51] Larsen S, Ara I, Rabøl R, Andersen JL, Boushel R, Dela F (2009). Are substrate use during exercise and mitochondrial respiratory capacity decreased in arm and leg muscle in type 2 diabetes?. Diabetologia.

[52] Mogensen M, Vind BF, Højlund K, Beck-Nielsen H, Sahlin K (2009). Maximal lipid oxidation in patients with type 2 diabetes is normal and shows an adequate increase in response to aerobic training. Diabetes Obes Metab.

[53] O'Connor E, Green S, Kiely C, O'Shea D, Egana M (2015). Differential effects of age and type 2 diabetes on dynamic vs. peak response of pulmonary oxygen uptake during exercise. J Appl Physiol (1985).

[54] O'Connor E, Kiely C, O'Shea D, Green S, Egana M (2012). Similar level of impairment in exercise performance and oxygen uptake kinetics in middle-aged men and women with type 2 diabetes. Am J Physiol Regul Integr Comp Physiol.

[55] Regensteiner JG, Sippel J, McFarling ET, Wolfel EE, Hiatt WR (1995). Effects of non-insulin-dependent diabetes on oxygen consumption during treadmill exercise. Med Sci Sports Exerc.

[56] Regensteiner JG, Bauer TA, Reusch JE, Brandenburg SL, Sippel JM, Vogelsong AM (1998). Abnormal oxygen uptake kinetic responses in women with type II diabetes mellitus. J Appl Physiol (1985).

[57] Regensteiner JG, Bauer TA, Reusch JE, Quaife RA, Chen MY, Smith SC (2009). Cardiac dysfunction during exercise in uncomplicated type 2 diabetes. Med Sci Sports Exerc.

[58] Wilson GA, Wilkins GT, Cotter JD, Lamberts RR, Lal S, Baldi JC (2017). Impaired ventricular filling limits cardiac reserve during submaximal exercise in people with type 2 diabetes. Cardiovasc Diabetol.

[59] IJzerman TH, Schaper NC, Melai T, Meijer K, Willems PJ, Savelberg HH (2012). Lower extremity muscle strength is reduced in people with type 2 diabetes, with and without polyneuropathy, and is associated with impaired mobility and reduced quality of life. Diabetes Res Clin Pract.

[60] Bauer TA, Reusch JE, Levi M, Regensteiner JG (2007). Skeletal muscle deoxygenation after the onset of moderate exercise suggests slowed microvascular blood flow kinetics in type 2 diabetes. Diabetes Care.

[61] van Tienen FH, Praet SF, de Feyter HM, van den Broek NM, Lindsey PJ, Schoonderwoerd KG (2012). Physical activity is the key determinant of skeletal muscle mitochondrial function in type 2 diabetes. J Clin Endocrinol Metab.

[62] Ozdirenç M, Biberoğlu S, Ozcan A (2003). Evaluation of physical fitness in patients with Type 2 diabetes mellitus. Diabetes Res Clin Pract.

[63] Zbinden-Foncea H, van Loon LJ, Raymackers JM, Francaux M, Deldicque L (2013). Contribution of nonesterified fatty acids to mitogen-activated protein kinase activation in human skeletal muscle during endurance exercise. Int J Sport Nutr Exerc Metab.

[64] Heberle I, Tonelli DC, Benedetti TB, Delevatti RS (2021). Similar functional capacity and handgrip strength of trained elderly women with and without type 2 diabetes mellitus: A cross-sectional study. Complement Ther Clin Pract.

[65] Tadic M, Suzic-Lazic J, Vukomanovic V, Cuspidi C, Ilic S, Celic V (2021). Functional capacity and left ventricular diastolic function in patients with type 2 diabetes. Acta Diabetol.

[66] Bassett DR, Howley ET (2000). Limiting factors for maximum oxygen uptake and determinants of endurance performance. Med Sci Sports Exerc.

[67] Oberbach A, Bossenz Y, Lehmann S, Niebauer J, Adams V, Paschke R (2006). Altered fiber distribution and fiber-specific glycolytic and oxidative enzyme activity in skeletal muscle of patients with type 2 diabetes. Diabetes Care.

[68] Schreuder TH, Maessen MF, Tack CJ, Thijssen DH, Hopman MT (2014). Life-long physical activity restores metabolic and cardiovascular function in type 2 diabetes. Eur J Appl Physiol.

[69] Segerström ÅB, Elgzyri T, Eriksson KF, Groop L, Thorsson O, Wollmer P (2011). Exercise capacity in relation to body fat distribution and muscle fibre distribution in elderly male subjects with impaired glucose tolerance, type 2 diabetes and matched controls. Diabetes Res Clin Pract.

[70] Yu TY, Jee JH, Bae JC, Hong WJ, Jin SM, Kim JH (2016). Delayed heart rate recovery after exercise as a risk factor of incident type 2 diabetes mellitus after adjusting for glycometabolic parameters in men. Int J Cardiol.

[71] Pelsers MM, Tsintzas K, Boon H, Jewell K, Norton L, Luiken JJ (2007). Skeletal muscle fatty acid transporter protein expression in type 2 diabetes patients compared with overweight, sedentary men and age-matched, endurance-trained cyclists. Acta Physiol (Oxf).

[72] Zierath JR, He L, Gumà A, Odegoard Wahlström E, Klip A, Wallberg-Henriksson H (1996). Insulin action on glucose transport and plasma membrane GLUT4 content in skeletal muscle from patients with NIDDM. Diabetologia.

[73] Nolen-Doerr E, Crick K, Saha C, de Groot M, Pillay Y, Shubrook JH (2018). Six-Minute Walk Test as a Predictive Measure of Exercise Capacity in Adults with Type 2 Diabetes. Cardiopulm Phys Ther J.

[74] Gremeaux V, Troisgros O, Benaïm S, Hannequin A, Laurent Y, Casillas JM (2011). Determining the minimal clinically important difference for the six-minute walk test and the 200-meter fast-walk test during cardiac rehabilitation program in coronary artery disease patients after acute coronary syndrome. Arch Phys Med Rehabil.

[75] Polkey MI, Spruit MA, Edwards LD, Watkins ML, Pinto-Plata V, Vestbo J (2013). Six-minute-walk test in chronic obstructive pulmonary disease: minimal clinically important difference for death or hospitalization. Am J Respir Crit Care Med.

[76] Barry VW, Baruth M, Beets MW, Durstine JL, Liu J, Blair SN (2014). Fitness vs. fatness on all-cause mortality: a meta-analysis. Prog Cardiovasc Dis.

[77] Wei M, Kampert JB, Barlow CE, Nichaman MZ, Gibbons LW, Paffenbarger RS (1999). Relationship between low cardiorespiratory fitness and mortality in normal-weight, overweight, and obese men. JAMA.

[78] Church TS, LaMonte MJ, Barlow CE, Blair SN (2005). Cardiorespiratory fitness and body mass index as predictors of cardiovascular disease mortality among men with diabetes. Arch Intern Med.

[79] Andrade-Mayorga O, Mancilla R, Díaz E, Alvarez C (2020). Heart Rate During an Exercise Test and Acute High-intensity Interval Training in Type 2 Diabetes. Int J Sports Med.

[80] Baldi JC, Aoina JL, Whalley GA, Carrick-Ranson G, Walsh HA, O'Shaughnessy H (2006). The effect of type 2 diabetes on diastolic function. Med Sci Sports Exerc.

[81] Chance WW, Rhee C, Yilmaz C, Dane DM, Pruneda ML, Raskin P (2008). Diminished alveolar microvascular reserves in type 2 diabetes reflect systemic microangiopathy. Diabetes Care.

[82] Durrer C, Francois M, Neudorf H, Little JP (2017). Acute high-intensity interval exercise reduces human monocyte Toll-like receptor 2 expression in type 2 diabetes. Am J Physiol Regul Integr Comp Physiol.

[83] Fluckey JD, Hickey MS, Brambrink JK, Hart KK, Alexander K, Craig BW (1994). Effects of resistance exercise on glucose tolerance in normal and glucose-intolerant subjects. J Appl Physiol (1985).

[84] Fujii N, Meade RD, Akbari P, Louie JC, Alexander LM, Boulay P (2017). No effect of ascorbate on cutaneous vasodilation and sweating in older men and those with type 2 diabetes exercising in the heat. Physiol Rep.

[85] Groen MB, Knudsen TA, Finsen SH, Pedersen BK, Hellsten Y, Mortensen SP (2019). Reduced skeletal-muscle perfusion and impaired ATP release during hypoxia and exercise in individuals with type 2 diabetes. Diabetologia.

[86] Gulsin GS, Swarbrick DJ, Athithan L, Brady EM, Henson J, Baldry E (2020). Effects of Low-Energy Diet or Exercise on Cardiovascular Function in Working-Age Adults With Type 2 Diabetes: A Prospective, Randomized, Open-Label, Blinded End Point Trial. Diabetes Care.

[87] Karavelioglu Y, Karapinar H, Gul İ, Kucukdurmaz Z, Yilmaz A, Akpek M (2013). Blood pressure response to exercise is exaggerated in normotensive diabetic patients. Blood Press.

[88] Meneilly GS, Elliott T, Tessier D, Hards L, Tildesley H (1996). NIDDM in the elderly. Diabetes Care.

[89] Meneilly GS, Elliott T (1999). Metabolic alterations in middle-aged and elderly obese patients with type 2 diabetes. Diabetes Care.

[90] Pinna V, Doneddu A, Roberto S, Magnani S, Ghiani G, Mulliri G (2021). Combined mental task and metaboreflex impair cerebral oxygenation in patients with type 2 diabetes mellitus. Am J Physiol Regul Integr Comp Physiol.

[91] Scheede-Bergdahl C, Benee Olsen D, Reving D, Boushel R, Dela F (2009). Cardiovascular disease markers in type 2 diabetes: the effects of a moderate home-based exercise training programme. Diab Vasc Dis Res.

[92] Suk MH, Moon YJ, Park SW, Park CY, Shin YA (2015). Maximal Fat Oxidation Rate during Exercise in Korean Women with Type 2 Diabetes Mellitus. Diabetes Metab J.

[93] Tobin LW, Kiens B, Galbo H (2008). The effect of exercise on postprandial lipidemia in type 2 diabetic patients. Eur J Appl Physiol.

[94] Vind BF, Pehmøller C, Treebak JT, Birk JB, Hey-Mogensen M, Beck-Nielsen H (2011). Impaired insulin-induced site-specific phosphorylation of TBC1 domain family, member 4 (TBC1D4) in skeletal muscle of type 2 diabetes patients is restored by endurance exercise-training. Diabetologia.

[95] Vukomanovic V, Suzic-Lazic J, Celic V, Cuspidi C, Grassi G, Galderisi M (2020). Is there association between left atrial function and functional capacity in patients with uncomplicated type 2 diabetes?. Int J Cardiovasc Imaging.

[96] Vukomanovic V, Suzic-Lazic J, Celic V, Cuspidi C, Petrovic T, Grassi G (2019). The relationship between functional capacity and left ventricular strain in patients with uncomplicated type 2 diabetes. J Hypertens.

[97] Vukomanovic V, Suzic-Lazic J, Celic V, Cuspidi C, Petrovic T, Ilic S (2019). Association between functional capacity and heart rate variability in patients with uncomplicated type 2 diabetes. Blood Press.

[98] Awotidebe TO, Adedoyin RA, Yusuf AO, Mbada CE, Opiyo R, Maseko FC (2014). Comparative functional exercise capacity of patients with type 2-diabetes and healthy controls: a case control study. Pan Afr Med J.

[99] Awotidebe TO, Ativie RN, Oke KI, Akindele MO, Adedoyin RA, Olaogun MO (2016). Relationships among exercise capacity, dynamic balance and gait characteristics of Nigerian patients with type-2 diabetes: an indication for fall prevention. J Exerc Rehabil.

[100] Hemmingsson E, Väisänen D, Andersson G, Wallin P, Ekblom-Bak E (2022). Combinations of BMI and cardiorespiratory fitness categories: trends between 1995 and 2020 and associations with CVD incidence and mortality and all-cause mortality in 471 216 adults. Eur J Prev Cardiol.

